# Crystal structures and Hirshfeld surface analysis of a series of 4-*O*-aryl­perfluoro­pyridines

**DOI:** 10.1107/S2056989019009344

**Published:** 2019-07-04

**Authors:** Andrew J. Peloquin, Cynthia A. Corley, Sonya K. Adas, Gary J. Balaich, Scott T. Iacono

**Affiliations:** a Department of Chemistry & Chemistry Research Center, United States Air Force, Academy, Colorado Springs, CO 80840, USA

**Keywords:** crystal structure, perfluoro­pyridine

## Abstract

Five new crystal structures of perfluoro­pyridine substituted in the 4-position with phen­oxy, 4-bromo­phen­oxy, naphthalen-2-yl­oxy, 6-bromo­naphthalen-2-yl­oxy, and 4,4′-biphen­oxy are reported, *viz.* 2,3,5,6-tetra­fluoro-4-phen­oxy­pyridine, C_11_H_5_F_4_NO (**I**), 4-(4-bromo­phen­oxy)-2,3,5,6-tetra­fluoro­pyridine, C_11_H_4_BrF_4_NO (**II**), 2,3,5,6-tetra­fluoro-4-[(naphthalen-2-yl)­oxy]pyridine, C_15_H_7_F_4_NO (**III**), 4-[(6-bromo­naphthalen-2-yl)­oxy]-2,3,5,6-tetra­fluoropyridine, C_15_H_6_BrF_4_NO (**IV**), and 2,2′-bis­[(perfluoro­pyridin-4-yl)­oxy]-1,1′-biphenyl, C_22_H_8_F_8_N_2_O_2_ (**V**). The dihedral angles between the aromatic ring systems in **I**–**IV** are 78.74 (8), 56.35 (8), 74.30 (7), and 64.34 (19)°, respectively. The complete mol­ecule of **V** is generated by a crystallographic twofold axis: the dihedral angle between the pyridine ring and adjacent phenyl ring is 80.89 (5)° and the equivalent angle between the biphenyl rings is 27.30 (5)°. In each crystal, the packing is driven by C—H⋯F inter­actions, along with a variety of C—F⋯π, C—H⋯π, C—Br⋯N, C—H⋯N, and C—Br⋯π contacts. Hirshfeld surface analysis was conducted to aid in the visualization of these various influences on the packing.

## Abstract 2

In each crystal of the title compounds the packing is driven by C—H⋯F inter­tactions, along with a variety of C—F⋯π, C—H⋯π, C—Br⋯N, C—H⋯N, and C—Br⋯π contacts. Hirshfeld surface analysis was conducted to aid in the visualization of these various influences on the packing.

## Chemical context   

Penta­fluoro­pyridine, or perfluoro­pyridine (C_5_F_5_N) is one of the most important perfluoro­heteroaromatic compounds. It is commercially available and its chemistry is well understood. As a result of the presence of five fluorine atoms, in addition to the nitro­gen atom of the pyridine ring, these systems are highly electrophilic and undergo substitution reactions readily in a predictable pattern (Baker & Muir, 2010 ▸; Chambers *et al.*, 1988 ▸). This chemistry has already been used in the design of several drugs (Bhambra *et al.*, 2016 ▸) and in peptide modification (Gimenez *et al.*, 2017 ▸). In an effort to further understand the inter­molecular inter­actions in the solid state of these fluorinated compounds, five new crystal structures of penta­fluoro­pyridine derivatives are herein reported as well as their syntheses.
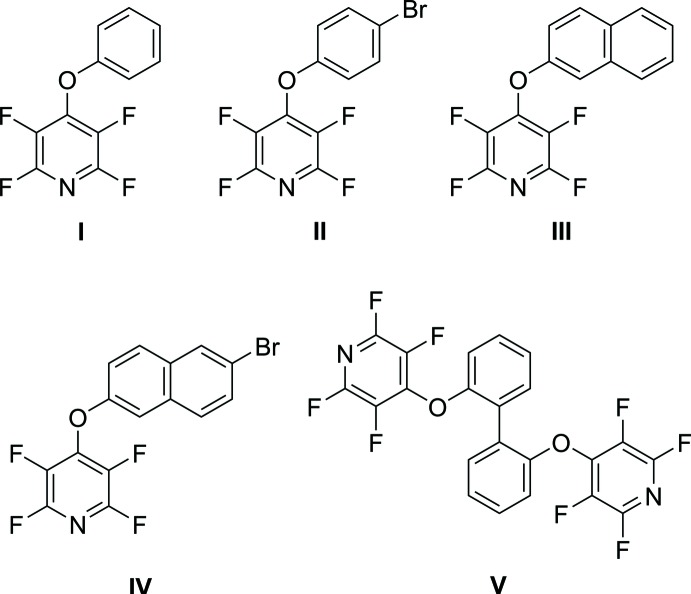


## Structural commentary   

**Figure 1:**
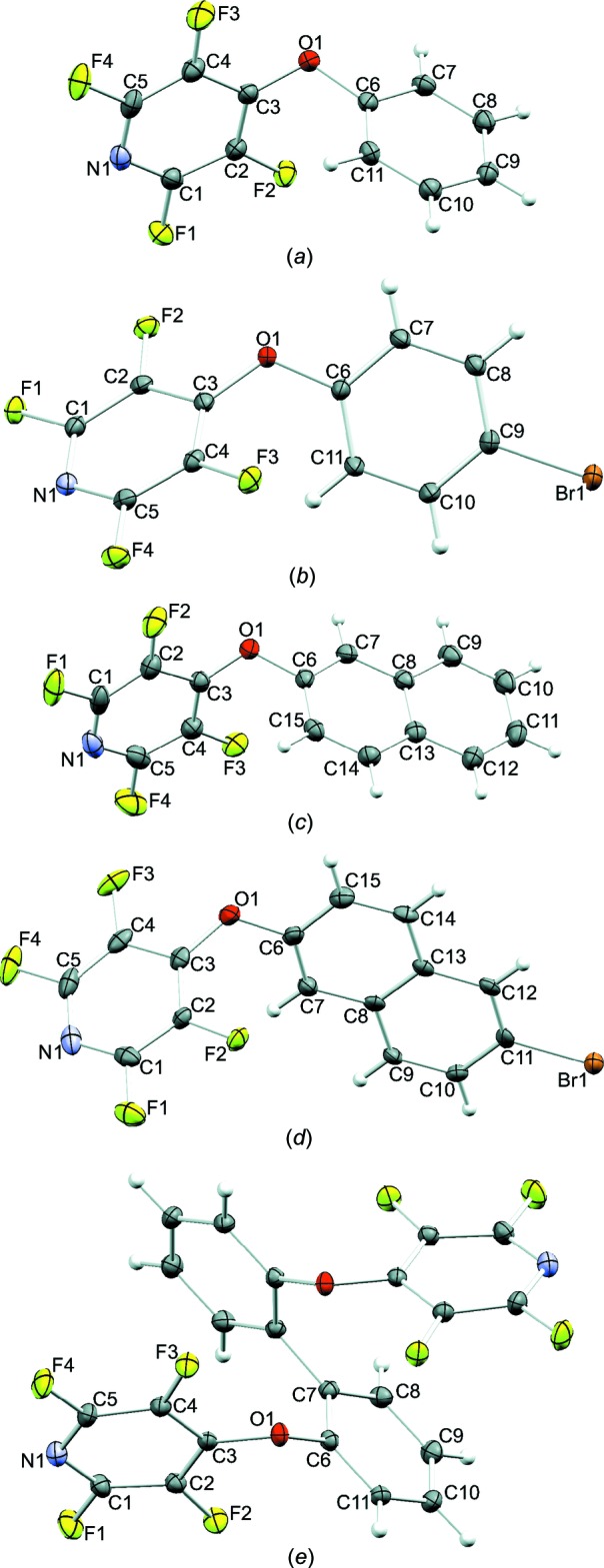
The mol­ecular structures of (*a*) **I**, (*b*) **II**, (*c*) **III**, (*d*) **IV**, and (*e*) **V**. Displacement ellipsoids are shown at the 50% probability level.

Compounds **I**, **III**, and **V** each crystallize in ortho­rhom­bic space groups, with **I** and **III** in *P*2_1_2_1_2_1_ and **V** in *Pbcn*. Compounds **II** and **IV** crystallize in the monoclinic space groups *P*2_1_/*n* and *P*2_1_ respectively (Fig. 1 ▸). With the exception of **V**, which has one half mol­ecule per asymmetric unit, each compound crystallizes with one mol­ecule per asymmetric unit. The dihedral angle between the aryl susbstituent and the pyridine ring ranges between 56.35 (8) and 80.89 (5)°. In **V**, the rings of the biphenyl system are rotated by 27.30 (5)° from each other.

## Supra­molecular features and Hirshfeld surface analysis   

In the crystal structures of each compound, the packing is consolidated by various C—H⋯F and C—F⋯π inter­actions (Tables 1 ▸–5 ▸ ▸ ▸ ▸). In **II**, the packing is aided by C—H⋯π and C—Br⋯N inter­actions, the latter of which lead to the formation of chains in the [10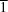] direction. Compound **III** also shows C—H⋯N hydrogen bonding. Further, in **IV**, C—H⋯π and C—Br⋯π inter­actions also contribute to the packing. Finally, in **I**, **III**, and **IV**, the packing is aided by halogen bonds of the type C—F⋯F—C or C—F⋯Br—C. In **I**, the C2—F2⋯F3 distance is 2.8156 (15) Å, with an angle of 119.54 (8)° (symmetry code: 1 − *x*, 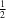 + *y*, 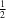 − *z*). For **III**, the C2—F2⋯F3 distance is 2.766 (15) Å, with an angle of 146.28 (10)° (symmetry code: 1 − *x*, 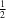 + *y*, 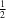 − *z*). The bromine atom participates in the halogen bonding of **IV**, with the C11—Br1⋯F2 distance being 3.095 (5) Å and the angle 164.46 (14)° (symmetry code: 2 − *x*, −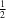 + *y*, 1 − *z*). All the observed halogen-bonding geometries fall within typically observed values (Cavallo *et al.*, 2016 ▸).

**Figure 2:**
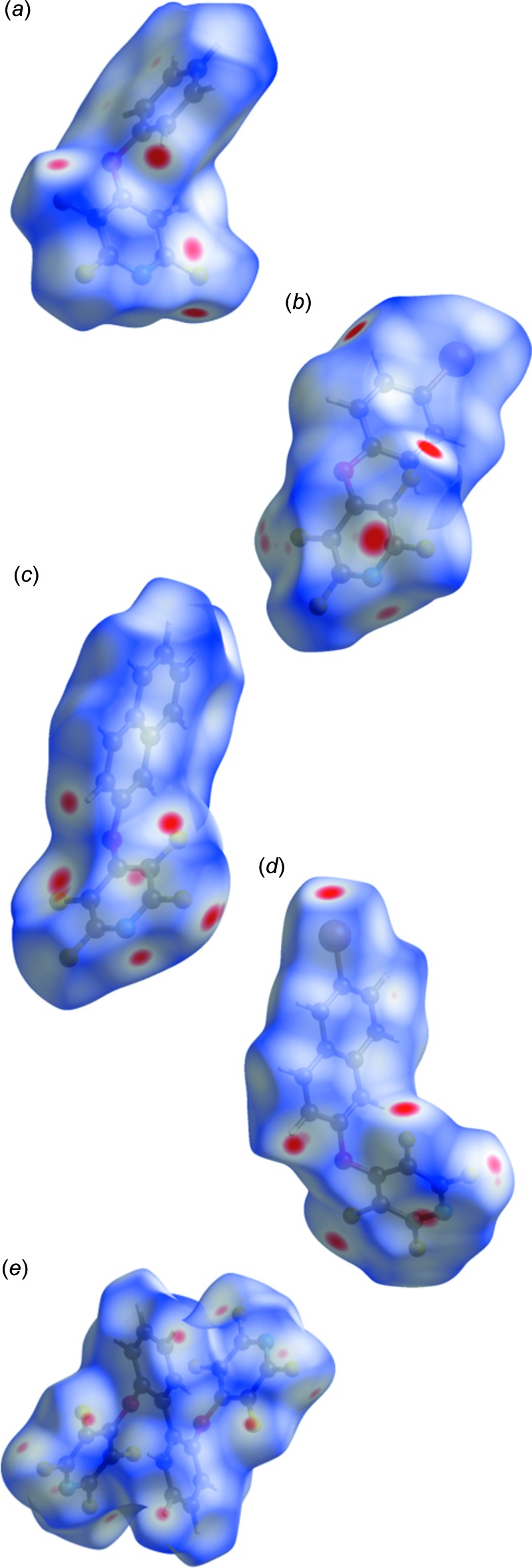
Hirshfeld surface of (*a*) **I**, (*b*) **II**, (*c*) **III**, (*d*) **IV**, and (*e*) **V** mapped with *d*_norm_.

Hirshfeld surface analysis was used to further investigate the inter­molecular inter­actions in the crystal structures. The Hirshfeld surface analysis (Spackman & Jayatilaka, 2009 ▸) was generated by *CrystalExplorer17.5* (Turner *et al.*, 2017 ▸), and was comprised of *d*_norm_ surface plots. The plots of the Hirshfeld surface are mapped over *d*_norm_ using standard surface resolution with a fixed colour scale of −0.1300 (red) to 1.2500 (blue). The characteristic bright-red spots near F1 and H11 in the Hirshfeld surface of **I** (Fig. 2 ▸ *a*) confirm the previously mentioned C11—H11⋯F1 (symmetry code: −*x* + 2, *y* − 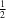, −*z* + 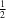) inter-atomic contacts. As expected, the same bright-red spots are observed for the C10—H10⋯F3 (symmetry code: −*x* + 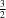, *y* + 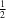, −*z* + 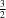) in **II** (Fig. 2 ▸ *b*), C7—H7⋯F3 (symmetry code: *x* + 1, *y*, *z*) in **III** (Fig. 2 ▸ *c*), C15—H15⋯F3 (symmetry code: −*x* + 1, *y* + 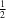, −*z* + 1) in **IV** (Fig. 2 ▸ *d*), and both C11—H11⋯F3^i^ and C11—H11⋯F3^ii^ [symmetry codes: (i) −*x* + 1, −*y* + 1, −*z* + 1; (ii) *x*, −*y* + 1, *z* − 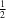] inter-atomic contacts in **V** (Fig. 2 ▸ *e*). The contributions of the various inter­molecular inter­actions in each of the title compounds are shown in Tables 6 ▸–10 ▸ ▸ ▸ ▸. With the exception of **IV**, the packing is dominated by ⋯H/H⋯F inter­actions, accounting for as high as 36.9% of the packing forces in **I**. In **IV**, the largest contribution is made by C⋯H/H⋯C inter­actions (19.1%), followed closely by F⋯H/H⋯F (18.8%).

Owing to the presence of nitro­gen, oxygen and bromine atoms in the various structures, several other contact types are confirmed by the Hirshfeld surface maps. In **II**, the C9—Br1⋯N1 (symmetry code: 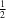 − *x*, 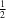 − *y*, −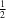 + *z*) halogen bond is clearly visible. The analogous halogen bond is not observed in **IV**. Only in **III** does a C—H⋯N inter­action significantly contribute to the packing, with the C15—H15⋯N1 (symmetry code: −*x*, *y* + 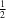, −*z* + 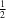] visible in the *d*_norm_ surface plot.

## Database survey   

A search of the November 2019 release of the Cambridge Structure Database (Groom *et al.*, 2016 ▸), with updates through May 2019, was performed using the program *ConQuest* (Bruno *et al.*, 2002 ▸). The search was limited to organic structures with *R* ≤ 0.1. A search for perfluoro­pyridines bearing a ether-linked substituents in the 4-position returned six results: 2,3,5,6-tetra­fluoro­pyridin-4-ol (UDUXEY; Sen *et al.*, 2009 ▸); benzo­phenone *O*-(2,3,5,6-tetra­fluoro-4-pyrid­yl)oxime (HICBAW; Banks *et al.*, 1995 ▸); methyl *N*,*O*-bis­(2,3,5,6-tetra­fluoro­pyridin-4-yl)threoninate (GOFCIP; Webster *et al.*, 2014 ▸); 2,3,5,6-tetra­fluoro-4-(4-nitro­phen­oxy)pyridine and 4,4′-[(1,1′-bi­naphthalene)-2,2′diylbis(­oxy)]bis­(tetra­fluoro­pyridine) (FISJUP and FISJOJ; Brittain & Cobb, 2019 ▸). In the bi­naphthalene-derived compound (FISJOJ), analogous to **V**, the naphthalene ring systems are rotated by 38.91 (5)° from each other.

## Synthesis and crystallization   

**2,3,5,6-Tetra­fluoro-4-phen­oxy­pyridine (I):** To a stirred solution of potassium carbonate (1 *M*, 147.5 ml), phenol (5.58 g, 59.0 mmol), penta­fluoro­pyridine (6.5 ml, 59 mmol), and DMF (150 ml) were added. The resulting solution was allowed to stir at room temperature for 24 h. Di­chloro­methane (75 ml) and saturated aqueous ammonium chloride (100 ml) were added and the biphasic solution stirred vigorously for an additional 24 h. The organic layer was separated, washed with water (5 × 200 ml), dried over MgSO_4_, and solvent removed *via* rotary evaporation. The resulting pale-brown solid was dissolved in refluxing EtOH (75 ml) and cooled to 278 K for 12 h. Vacuum filtration, washing with cold EtOH (20 ml) and vacuum drying afforded the target compound as a white, crystalline solid (14.3 g, 99%). Colourless needles were obtained from a saturated EtOH solution by cooling to 298 K. ^1^H NMR (400 MHz, CDCl_3_): 6.92 (*d*, 2H, *J* = 8.0 Hz), 7.08 (*d*, 1H, *J* = 8.4 Hz), 7.24 (*t*, 2H, *J* = 8.0 Hz). ^19^F NMR (376 MHz, CDCl_3_): −88.9, −154.4.

**4-(4-Bromo­phen­oxy)-2,3,5,6-tetra­fluoro­pyridine (II):** To a stirred solution of potassium carbonate (1 *M*, 22.8 ml), 4-bromo­phenol (1.58 g, 9.11 mmol), penta­fluoro­pyridine (1.00 ml, 9.11 mmol), and DMF (25 ml) were added. The resulting solution was allowed to stir at room temperature for 24 h. Diethyl ether (50 ml) and saturated aqueous ammonium chloride (50 ml) were added and the biphasic solution stirred vigorously for an additional 24 h. The organic layer was separated, washed with water (5 × 100 ml), dried over MgSO_4_, and solvent removed *via* rotary evaporation. The resulting pale brown solid was dissolved in refluxing EtOH (15 ml) and cooled to 278 K for 12 h. Vacuum filtration, washing with cold EtOH (20 ml) and vacuum drying afforded the target compound as a white, crystalline solid (2.12 g, 73%). Colourless rectangular prisms were obtained from a saturated EtOH solution by cooling to 298 K. ^1^H NMR (400 MHz, CDCl_3_): 7.50 (*d*, 2H, *J* = 7.5 Hz), 6.96 (*d*, 2H, *J* = 7.5 Hz). ^19^F NMR (376 MHz, CDCl_3_): −87.9, −154.0.

**2,3,5,6-Tetra­fluoro-4-[(naphthalen-2-yl)­oxy]pyridine (III):** To a stirred solution of potassium carbonate (1 *M*, 90 ml), naphthalen-2-ol (4.98 g, 34.5 mmol), penta­fluoro­pyridine (3.8 ml, 34.5 mmol), and DMF (100 ml) were added. The resulting solution was allowed to stir at room temperature for 24 h. Di­chloro­methane (50 ml) and saturated aqueous ammonium chloride (100 ml) were added and the biphasic solution stirred vigorously for an additional 24 h. The organic layer was separated, washed with water (5 × 200 ml), dried over MgSO_4_, and solvent removed *via* rotary evaporation. The resulting pale brown solid was dissolved in refluxing EtOH (50 ml) and cooled to 298 K for 12 h. Vacuum filtration, washing with cold EtOH (20 ml) and vacuum drying afforded the target compound as a white, crystalline solid (5.90 g, 58%). Colourless rectangular prisms were obtained from a saturated EtOH solution by cooling to 298 K. ^1^H NMR (500 MHz, CDCl_3_): 7.90-7.86 (*m*, 2H), 7.76 (*d*, 1H, *J* = 8 Hz), 7.54-7.47 (*m*, 2H), 7.35–7.31 (*m*, 2H). ^19^F NMR (471 MHz, CDCl_3_): −88.3, −154.0.

**2,3,5,6-Tetra­fluoro-4-[(6-bromo­naphthalen-2-yl)­oxy]pyri­dine (IV):** To a stirred solution of potassium carbon­ate (1 *M*, 60 ml), 6-bromo-2-naphthol (5.00 g, 22.4 mmol), penta­fluoro­pyridine (2.45 ml, 22.4 mmol), and DMF (60 ml) were added. The resulting solution was allowed to stir at room temperature for 24 h. Diethyl ether (50 ml) and saturated aqueous ammonium chloride (100 ml) were added and the biphasic solution stirred vigorously for an additional 2 h. The organic layer was separated, washed with water (5 × 200 ml), dried over MgSO_4_, and solvent removed *via* rotary evaporation. The resulting off-white solid was dissolved in refluxing EtOH (40 ml) and cooled to 298 K for 12 h. Vacuum filtration, washing with cold EtOH (20 ml) and vacuum drying afforded the target compound as a white, crystalline solid (6.46 g, 78%). Colourless plates were obtained from a saturated EtOH solution by cooling to 298 K. ^1^H NMR (500 MHz, CDCl_3_): 8.00 (*s*, 1H), 7.81–7.75 (*m*, 1H), 7.63–7.55 (*m*, 2H), 7.35–7.27 (*m*, 2H). ^19^F NMR (471 MHz, CDCl_3_): −87.9, −153.8.

**2,2′-Bis[(2,3,5,6-tetra­fluoro­pyridin-4-yl)­oxy]-1,1′-biphenyl (V):** To a stirred solution of potassium carbonate (1 *M*, 30 ml), 2,2′-biphenol (1.02 g, 5.37 mmol), penta­fluoro­pyridine (1.2 ml, 11 mmol), and DMF (30 ml) were added. The resulting solution was allowed to stir at room temperature for 24 h. Diethyl ether (50 ml) and saturated aqueous ammonium chloride (100 ml) were added and the biphasic solution stirred vigorously for an additional 2 h. The organic layer was separated, washed with water (5 × 200 ml), dried over MgSO_4_, and solvent removed *via* rotary evaporation. The resulting off-white solid was dissolved in refluxing EtOH (10 ml) and cooled to 298 K for 12 h. Vacuum filtration, washing with cold EtOH (20 ml) and vacuum drying afforded the target compound as a white solid (2.57 g, 97%). Colourless rectangular prisms were obtained from a saturated EtOH solution by cooling to 298 K. ^1^H NMR (500 MHz, CDCl_3_): 7.45–7.24 (*m*, 6H), 6.99 (*t*, 2H, *J* = 7.5 Hz). ^19^F NMR (471 MHz, CDCl_3_): −88.9, −155.0.

## Refinement   

Crystal data, data collection and structure refinement details are summarized in Table 11 ▸. H atoms were positioned geometrically and refined using a riding model with C—H = 0.95 Å and *U*_iso_(H) = 1.2*U*_eq_(C). The absolute structures of **I** and **III** were inter­mediate in the present refinement. Compound **IV** was refined as an inversion twin.

## Supplementary Material

Crystal structure: contains datablock(s) global, I, II, III, IV, V. DOI: 10.1107/S2056989019009344/hb7833sup1.cif

Structure factors: contains datablock(s) I. DOI: 10.1107/S2056989019009344/hb7833Isup2.hkl

Supporting information file. DOI: 10.1107/S2056989019009344/hb7833Isup7.cml

Structure factors: contains datablock(s) II. DOI: 10.1107/S2056989019009344/hb7833IIsup3.hkl

Supporting information file. DOI: 10.1107/S2056989019009344/hb7833IIsup8.cml

Structure factors: contains datablock(s) III. DOI: 10.1107/S2056989019009344/hb7833IIIsup4.hkl

Supporting information file. DOI: 10.1107/S2056989019009344/hb7833IIIsup9.cml

Supporting information file. DOI: 10.1107/S2056989019009344/hb7833IVsup10.cml

Structure factors: contains datablock(s) IV. DOI: 10.1107/S2056989019009344/hb7833IVsup5.hkl

Supporting information file. DOI: 10.1107/S2056989019009344/hb7833Vsup11.cml

Structure factors: contains datablock(s) V. DOI: 10.1107/S2056989019009344/hb7833Vsup6.hkl

CCDC references: 1937619, 1937620, 1937621, 1937622, 1937623

Additional supporting information:  crystallographic information; 3D view; checkCIF report

## Figures and Tables

**Table 1:** Contact geometry (Å, °) for **I** *Cg*1 is the centroid of the N1/C1–C5 ring.

*X—Y⋯A*	*X—Y*	*Y⋯A*	*X⋯A*	*X—Y⋯A*
C11—H11⋯F1^i^	0.95	2.46	3.4049 (19)	170.3
C1—F1⋯*Cg*1^ii^	1.3404 (18)	3.6822 (13)	4.8632 (17)	147.20 (9)

Symmetry codes: (i) −*x* + 2, *y* − 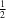, −*z* + 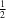; (ii) 2 − *x*, 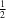 + *x*, 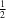 − *z*.

**Table 2:** Contact geometry (Å, °) for **II** *Cg*1 and *Cg*2 are the centroids of the N1/C1–C4 and C6–C11 rings, respectively.

*X—Y⋯A*	*X—Y*	*Y⋯A*	*X⋯A*	*X—Y⋯A*
C10—H10⋯F3^i^	0.95	2.39	3.2214 (19)	145.9
C11—H11⋯*Cg*2^i^	0.95	2.90	3.668 (2)	139
C1—F1⋯*Cg*1^ii^	1.3395 (19)	3.1531 (14)	4.068 (2)	124.76 (10)
C5—F4⋯*Cg*1^iii^	1.3401 (19)	3.1094 (13)	3.6241 (18)	101.55 (9)
C9—Br1⋯N1^iv^	1.8952 (17)	3.2639 (16)	5.104 (3)	162.65 (7)

Symmetry codes: (i) −*x* + 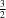, *y* + 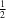, −*z* + 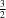; (ii) −*x* + 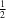, *y* + 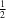, −*z* + 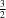; (iii) −*x* + 1, −*y* + 1, −*z* + 2; (iv) 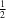 + *x*, 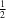 − *y*, −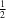 + *z*.

**Table 3:** Contact geometry (Å, °) for **III** *Cg*1 and *Cg*2 are the centroids of the N1/C1–C5 and C6–C15 rings, respectively.

*X—Y⋯A*	*X—Y*	*Y⋯A*	*X⋯A*	*X—Y⋯A*
C7—H7⋯F3^i^	0.95	2.50	3.4366 (19)	169.6
C15—H15⋯N1^ii^	0.95	2.59	3.455 (2)	152.1
C1—F1⋯*Cg*2^iii^	1.334 (2)	3.2922 (15)	3.5581 (19)	90.28 (10)
C5—F4⋯*Cg*1^iv^	1.343 (2)	3.2790 (14)	4.2804 (18)	130.88 (11)

Symmetry codes: (i) *x* + 1, *y*, *z*; (ii) −*x*, *y* + 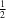, −*z* + 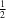; (iii) 1 − *x*, −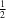 + *y*, 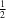 − *z*; (iv) − *x*, −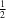 + *y*, 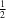 − *z*.

**Table 4:** Contact geometry (Å, °) for **IV** *Cg*1, *Cg*2, and *Cg*3 are the centroids of the N1/C1–C5, C6–C15, and C8–C13 rings, respectively.

*X—Y⋯A*	*X—Y*	*Y⋯A*	*X⋯A*	*X—Y⋯A*
C15—H15⋯F3^ii^	0.95	2.43	3.105 (5)	127.5
C9—H9⋯*Cg*3^i^	0.95	2.83	3.519 (5)	130
C11—Br1⋯*Cg*1^iii^	1.901 (4)	3.6283 (19)	4.923 (5)	122.74 (13)
C11—Br1⋯*Cg*2^iv^	1.901 (4)	3.735 (2)	5.037 (4)	123.34 (12)
C4—F3⋯*Cg*1^v^	1.344 (6)	3.082 (3)	3.936 (5)	120.3 (3)

Symmetry codes: (i) −*x* + 1, *y* + 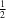, −*z* + 1; (ii) *x* + 1, *y*, *z*; (iii) −*x* + 1, *y* − 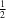, −*z* + 1; (iv) −*x* + 2, *y* − 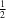, −*z* + 1; (v) −*x*, *y* − 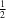, −*z*.

**Table 5:** Contact geometry (Å, °) for **V** *Cg*1 is the centroid of the N1/C1–C5 ring.

*X—Y⋯A*	*X—Y*	*Y⋯A*	*X⋯A*	*X—Y⋯A*
C11—H11⋯F3^i^	0.95	2.63	3.2361 (13)	121.8
C11—H11⋯F3^ii^	0.95	2.60	3.2711 (12)	127.8
C11—H11⋯O1^i^	0.95	2.61	3.3446 (13)	134.3
C5—F4⋯*Cg*1^iii^	1.3382 (12)	3.4138 (9)	4.3778 (12)	128.77 (6)

Symmetry codes: (i) −*x* + 1, −*y* + 1, −*z* + 1; (ii) *x*, −*y* + 1, *z* − 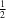; (iii) *x*, 1 − *y*, 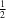 + *z*.

**Table 6:** Percentage contributions of inter-atomic contacts to the Hirshfeld surface for **I**

Contact	Percentage contribution
F⋯H/H⋯F	36.9
C⋯H/H⋯C	14.8
F⋯F	12.1
H⋯H	9.6
N⋯H/H⋯N	4.8
F⋯C/C⋯F	4.7
F⋯O/O⋯F	4.2
C⋯C	4.1
N⋯C/C⋯N	3.2
O⋯C/C⋯O	2.2
O⋯N/N⋯O	2.0
F⋯N/N⋯F	1.3
O⋯H/H⋯O	0.1

**Table 7:** Percentage contributions of inter-atomic contacts to the Hirshfeld surface for **II**

Contact	Percentage contribution
F⋯H/H⋯F	18.7
F⋯C/C⋯F	11.8
C⋯H/H⋯C	11.7
F⋯F	10.8
Br⋯F/F⋯Br	8.3
Br⋯H/H⋯Br	7.7
H⋯H	6.9
F⋯N/N⋯F	6.1
O⋯H/H⋯O	4.6
Br⋯N/N⋯Br	3.5
C⋯C	3.4
Br⋯C/C⋯Br	2.2
Br⋯O/O⋯Br	1.8
N⋯C/C⋯N	0.9
F⋯O/O⋯F	0.8
O⋯C/C⋯O	0.6
N⋯H/H⋯N	0.2

**Table 8:** Percentage contributions of inter-atomic contacts to the Hirshfeld surface for **III**

Contact	Percentage contribution
F⋯H/H⋯F	30.4
C⋯H/H⋯C	22.8
H⋯H	14.0
F⋯C/C⋯F	10.0
F⋯F	6.6
N⋯H/H⋯N	4.2
O⋯C/C⋯O	2.9
F⋯O/O⋯F	2.3
F⋯N/N⋯F	2.0
O⋯H/H⋯O	0.3
O⋯N/N⋯O	1.6
C⋯C	1.5
N⋯C/C⋯N	1.4

**Table 9:** Percentage contributions of inter-atomic contacts to the Hirshfeld surface for **IV**

Contact	Percentage contribution
C⋯H/H⋯C	19.1
F⋯H/H⋯F	18.8
F⋯C/C⋯F	9.4
H⋯H	9.1
Br⋯H/H⋯Br	8.7
F⋯F	7.7
Br⋯C/C⋯Br	7.2
F⋯O/O⋯F	4.5
Br⋯F/F⋯Br	3.9
F⋯N/N⋯F	3.7
N⋯H/H⋯N	2.6
C⋯C	1.5
N⋯C/C⋯N	1.1
O⋯N/N⋯O	0.9
Br⋯N/N⋯Br	0.8
O⋯C/C⋯O	0.6
O⋯H/H⋯O	0.3

**Table 10:** Percentage contributions of inter-atomic contacts to the Hirshfeld surface for **V**

Contact	Percentage contribution
F⋯H/H⋯F	32.3
F⋯C/C⋯F	19.0
H⋯H	11.6
F⋯F	11.3
N⋯H/H⋯N	7.1
F⋯N/N⋯F	6.3
C⋯H/H⋯C	4.8
O⋯H/H⋯O	4.5
F⋯O/O⋯F	1.8
C⋯C	1.3

**Table 11:** Experimental details

	**I**	**II**	**III**	**IV**	**V**
Crystal data					
Chemical formula	C_11_H_5_F_4_NO	C_11_H_4_BrF_4_NO	C_15_H_7_F_4_NO	C_15_H_6_BrF_4_NO	C_22_H_8_F_8_N_2_O_2_
*M*_r_	243.16	322.06	293.22	372.12	484.30
Crystal system, space group	Orthorhombic, *P*2_1_2_1_2_1_	Monoclinic, *P*2_1_/*n*	Orthorhombic, *P*2_1_2_1_2_1_	Monoclinic, *P*2_1_	Orthorhombic, *P* *b* *c* *n*
Temperature (K)	100	100	100	100	100
*a*, *b*, *c* (Å)	5.4199 (5), 10.3293 (9), 17.4076 (15)	13.3530 (5), 5.8584 (2), 14.8863 (6)	5.4703 (5), 9.2548 (9), 24.109 (2)	6.0135 (3), 7.4994 (4), 14.6318 (7)	18.8516 (6), 10.6512 (3), 9.2196 (3)
α, β, γ (°)	90, 90, 90	90, 113.585 (2), 90	90, 90, 90	90, 101.401 (2), 90	90, 90, 90
*V* (Å^3^)	974.54 (15)	1067.24 (7)	1220.6 (2)	646.84 (6)	1851.22 (10)
*Z*	4	4	4	2	4
Radiation type	Mo *K*α	Mo *K*α	Mo *K*α	Mo *K*α	Mo *K*α
μ (mm^−1^)	0.16	3.89	0.14	3.23	0.17
Crystal size (mm)	0.25 × 0.11 × 0.09	0.15 × 0.10 × 0.09	0.54 × 0.36 × 0.29	0.30 × 0.14 × 0.04	0.33 × 0.27 × 0.26

Data collection					
Diffractometer	Bruker SMART APEX CCD	Bruker SMART APEX CCD	Bruker SMART APEX CCD	Bruker SMART APEX CCD	Bruker SMART APEX CCD
Absorption correction	Multi-scan (*SADABS*; Bruker, 2017 ▸)	Multi-scan (*SADABS*; Bruker, 2017 ▸)	Multi-scan (*SADABS*; Bruker, 2017 ▸)	Multi-scan (*SADABS*; Bruker, 2017 ▸)	Multi-scan (*SADABS*; Bruker, 2017 ▸)
*T*_min_, *T*_max_	0.93, 0.99	0.21, 0.72	0.83, 0.96	0.63, 0.89	0.87, 0.96
No. of measured, independent and observed [*I* > 2σ(*I*)] reflections	13236, 2603, 2520	19174, 3544, 3013	26820, 3296, 3149	12954, 2775, 2566	36854, 2830, 2554
*R*_int_	0.021	0.040	0.025	0.040	0.028
(sin θ/λ)_max_ (Å^−1^)	0.684	0.735	0.684	0.641	0.714

Refinement					
*R*[*F* ^2^ > 2σ(*F* ^2^)], *wR*(*F* ^2^), *S*	0.028, 0.073, 1.05	0.031, 0.083, 1.06	0.031, 0.078, 1.07	0.031, 0.058, 1.14	0.036, 0.098, 1.06
No. of reflections	2603	3544	3296	2775	2830
No. of parameters	154	163	190	200	154
No. of restraints	0	0	0	1	0
H-atom treatment	H-atom parameters constrained	H-atom parameters constrained	H-atom parameters constrained	H-atom parameters constrained	H-atom parameters constrained
Δρ_max_, Δρ_min_ (e Å^−3^)	0.26, −0.15	0.84, −0.77	0.27, −0.15	0.70, −0.47	0.54, −0.24
Absolute structure	Flack *x* determined using 1019 quotients [(*I* ^+^)−(*I* ^−^)]/[(*I* ^+^)+(*I* ^−^)] (Parsons *et al.*, 2013 ▸)	–	Flack *x* determined using 1255 quotients [(*I* ^+^)−(*I* ^−^)]/[(*I* ^+^)+(*I* ^−^)] (Parsons *et al.*, 2013 ▸)	Refined as an inversion twin	–
Absolute structure parameter	−0.20 (13)	–	−0.08 (14)	0.171 (12)	–

Computer programs: *APEX3* and *SAINT* (Bruker, 2017 ▸), *SHELXT* (Sheldrick, 2015*a* ▸), *SHELXL2016/6* (Sheldrick, 2015*b* ▸), *Mercury* (Macrae *et al.*, 2008 ▸), *Olex2* (Dolomanov *et al.*, 2009 ▸) and *publCIF* (Westrip, 2010 ▸).

## supplementary crystallographic information

### 2,3,5,6-Tetrafluoro-4-phenoxypyridine (I) Crystal data

**Table:**

C_11_H_5_F_4_NO	*D*_x_ = 1.657 Mg m^−^^3^
*M**_r_* = 243.16	Mo *K*α radiation, λ = 0.71073 Å
Orthorhombic, *P*2_1_2_1_2_1_	Cell parameters from 7895 reflections
*a* = 5.4199 (5) Å	θ = 2.3–29.5°
*b* = 10.3293 (9) Å	µ = 0.16 mm^−^^1^
*c* = 17.4076 (15) Å	*T* = 100 K
*V* = 974.54 (15) Å^3^	Needle, colourless
*Z* = 4	0.25 × 0.11 × 0.09 mm
*F*(000) = 488	

### 2,3,5,6-Tetrafluoro-4-phenoxypyridine (I) Data collection

**Table:**

Bruker SMART APEX CCD diffractometer	2603 independent reflections
Radiation source: fine focus sealed tube	2520 reflections with *I* > 2σ(*I*)
Graphite monochromator	*R*_int_ = 0.021
Detector resolution: 8.3333 pixels mm^-1^	θ_max_ = 29.1°, θ_min_ = 2.3°
ω Scans scans	*h* = −7→7
Absorption correction: multi-scan (SADABS; Bruker, 2017)	*k* = −14→14
*T*_min_ = 0.93, *T*_max_ = 0.99	*l* = −23→23
13236 measured reflections	

### 2,3,5,6-Tetrafluoro-4-phenoxypyridine (I) Refinement

**Table:**

Refinement on *F*^2^	Hydrogen site location: inferred from neighbouring sites
Least-squares matrix: full	H-atom parameters constrained
*R*[*F*^2^ > 2σ(*F*^2^)] = 0.028	*w* = 1/[σ^2^(*F*_o_^2^) + (0.0371*P*)^2^ + 0.2471*P*] where *P* = (*F*_o_^2^ + 2*F*_c_^2^)/3
*wR*(*F*^2^) = 0.073	(Δ/σ)_max_ < 0.001
*S* = 1.05	Δρ_max_ = 0.26 e Å^−^^3^
2603 reflections	Δρ_min_ = −0.15 e Å^−^^3^
154 parameters	Absolute structure: Flack *x* determined using 1019 quotients [(*I*^+^)-(*I*^-^)]/[(*I*^+^)+(*I*^-^)] (Parsons *et al.*, 2013)
0 restraints	Absolute structure parameter: −0.20 (13)
Primary atom site location: structure-invariant direct methods	

### 2,3,5,6-Tetrafluoro-4-phenoxypyridine (I) Special details

**Table:**

Geometry. All esds (except the esd in the dihedral angle between two l.s. planes) are estimated using the full covariance matrix. The cell esds are taken into account individually in the estimation of esds in distances, angles and torsion angles; correlations between esds in cell parameters are only used when they are defined by crystal symmetry. An approximate (isotropic) treatment of cell esds is used for estimating esds involving l.s. planes.

### 2,3,5,6-Tetrafluoro-4-phenoxypyridine (I) Fractional atomic coordinates and isotropic or equivalent isotropic displacement parameters (Å^2^)

**Table:**

	*x*	*y*	*z*	*U*_iso_*/*U*_eq_	
F1	1.0458 (2)	0.96948 (10)	0.27358 (6)	0.0304 (2)	
F2	0.64334 (19)	0.87849 (9)	0.34737 (5)	0.0247 (2)	
F3	0.4522 (2)	0.64159 (11)	0.12257 (6)	0.0339 (3)	
F4	0.8628 (2)	0.74537 (11)	0.05818 (6)	0.0354 (3)	
O1	0.3304 (2)	0.70337 (11)	0.27133 (6)	0.0220 (2)	
N1	0.9535 (3)	0.85667 (14)	0.16598 (8)	0.0232 (3)	
C1	0.8951 (3)	0.88735 (15)	0.23682 (9)	0.0214 (3)	
C2	0.6915 (3)	0.84050 (14)	0.27546 (8)	0.0185 (3)	
C3	0.5371 (3)	0.75320 (15)	0.23844 (9)	0.0182 (3)	
C4	0.5954 (3)	0.72270 (15)	0.16263 (9)	0.0223 (3)	
C5	0.8047 (3)	0.77713 (16)	0.13038 (9)	0.0240 (3)	
C6	0.3557 (3)	0.65167 (14)	0.34628 (8)	0.0179 (3)	
C7	0.1718 (3)	0.68151 (16)	0.39875 (9)	0.0212 (3)	
H7	0.041504	0.738816	0.385477	0.025*	
C8	0.1829 (3)	0.62530 (16)	0.47149 (9)	0.0227 (3)	
H8	0.058655	0.644261	0.508309	0.027*	
C9	0.3749 (3)	0.54150 (15)	0.49061 (9)	0.0214 (3)	
H9	0.380741	0.503169	0.540181	0.026*	
C10	0.5583 (3)	0.51402 (15)	0.43697 (9)	0.0210 (3)	
H10	0.689859	0.4575	0.450189	0.025*	
C11	0.5492 (3)	0.56943 (15)	0.36367 (9)	0.0194 (3)	
H11	0.673132	0.550979	0.326676	0.023*	

### 2,3,5,6-Tetrafluoro-4-phenoxypyridine (I) Atomic displacement parameters (Å^2^)

**Table:**

	*U*^11^	*U*^22^	*U*^33^	*U*^12^	*U*^13^	*U*^23^
F1	0.0275 (5)	0.0317 (5)	0.0320 (5)	−0.0123 (4)	−0.0031 (4)	0.0032 (4)
F2	0.0311 (5)	0.0254 (5)	0.0177 (4)	−0.0052 (4)	0.0026 (4)	−0.0038 (4)
F3	0.0478 (7)	0.0312 (5)	0.0228 (5)	−0.0132 (5)	−0.0036 (5)	−0.0051 (4)
F4	0.0497 (7)	0.0354 (6)	0.0210 (5)	0.0030 (5)	0.0127 (5)	−0.0014 (4)
O1	0.0185 (5)	0.0281 (6)	0.0193 (5)	−0.0037 (5)	−0.0018 (4)	0.0049 (4)
N1	0.0218 (6)	0.0234 (6)	0.0242 (6)	0.0035 (5)	0.0047 (5)	0.0067 (5)
C1	0.0201 (7)	0.0196 (7)	0.0245 (7)	−0.0010 (6)	−0.0025 (6)	0.0044 (6)
C2	0.0208 (7)	0.0184 (6)	0.0163 (6)	0.0007 (6)	−0.0006 (5)	0.0013 (5)
C3	0.0178 (6)	0.0177 (6)	0.0191 (6)	0.0008 (5)	−0.0012 (5)	0.0029 (5)
C4	0.0295 (8)	0.0184 (7)	0.0190 (7)	−0.0002 (6)	−0.0018 (6)	0.0005 (5)
C5	0.0316 (8)	0.0222 (7)	0.0182 (7)	0.0061 (7)	0.0058 (6)	0.0029 (6)
C6	0.0178 (6)	0.0185 (6)	0.0175 (6)	−0.0035 (5)	−0.0013 (5)	0.0009 (5)
C7	0.0160 (6)	0.0235 (7)	0.0240 (7)	0.0018 (6)	0.0000 (6)	−0.0006 (6)
C8	0.0199 (7)	0.0270 (7)	0.0214 (7)	−0.0002 (6)	0.0041 (6)	−0.0017 (6)
C9	0.0238 (7)	0.0216 (7)	0.0188 (7)	−0.0013 (6)	0.0010 (6)	0.0019 (5)
C10	0.0199 (7)	0.0186 (7)	0.0245 (7)	0.0013 (6)	0.0005 (6)	0.0022 (6)
C11	0.0183 (6)	0.0186 (7)	0.0212 (7)	−0.0002 (5)	0.0035 (6)	0.0000 (5)

### 2,3,5,6-Tetrafluoro-4-phenoxypyridine (I) Geometric parameters (Å, º)

**Table:**

F1—C1	1.3404 (18)	C6—C11	1.383 (2)
F2—C2	1.3375 (17)	C6—C7	1.387 (2)
F3—C4	1.3382 (19)	C7—C8	1.394 (2)
F4—C5	1.3366 (18)	C7—H7	0.9500
O1—C3	1.3594 (18)	C8—C9	1.394 (2)
O1—C6	1.4164 (18)	C8—H8	0.9500
N1—C5	1.307 (2)	C9—C10	1.393 (2)
N1—C1	1.312 (2)	C9—H9	0.9500
C1—C2	1.380 (2)	C10—C11	1.399 (2)
C2—C3	1.389 (2)	C10—H10	0.9500
C3—C4	1.393 (2)	C11—H11	0.9500
C4—C5	1.385 (2)		
			
C3—O1—C6	116.81 (12)	C11—C6—O1	120.43 (13)
C5—N1—C1	116.66 (14)	C7—C6—O1	116.98 (13)
N1—C1—F1	117.05 (14)	C6—C7—C8	118.34 (15)
N1—C1—C2	124.49 (15)	C6—C7—H7	120.8
F1—C1—C2	118.46 (14)	C8—C7—H7	120.8
F2—C2—C1	120.62 (14)	C9—C8—C7	120.50 (15)
F2—C2—C3	120.48 (14)	C9—C8—H8	119.8
C1—C2—C3	118.90 (14)	C7—C8—H8	119.8
O1—C3—C2	123.16 (13)	C10—C9—C8	119.95 (14)
O1—C3—C4	120.02 (14)	C10—C9—H9	120.0
C2—C3—C4	116.73 (14)	C8—C9—H9	120.0
F3—C4—C5	121.20 (15)	C9—C10—C11	120.18 (15)
F3—C4—C3	120.24 (15)	C9—C10—H10	119.9
C5—C4—C3	118.56 (15)	C11—C10—H10	119.9
N1—C5—F4	117.04 (15)	C6—C11—C10	118.52 (14)
N1—C5—C4	124.62 (15)	C6—C11—H11	120.7
F4—C5—C4	118.33 (16)	C10—C11—H11	120.7
C11—C6—C7	122.50 (14)		
			
C5—N1—C1—F1	−179.60 (14)	C1—N1—C5—F4	180.00 (14)
C5—N1—C1—C2	0.7 (2)	C1—N1—C5—C4	−1.1 (2)
N1—C1—C2—F2	−178.92 (14)	F3—C4—C5—N1	−179.80 (15)
F1—C1—C2—F2	1.4 (2)	C3—C4—C5—N1	−0.4 (2)
N1—C1—C2—C3	1.1 (2)	F3—C4—C5—F4	−0.9 (2)
F1—C1—C2—C3	−178.63 (13)	C3—C4—C5—F4	178.56 (14)
C6—O1—C3—C2	−49.3 (2)	C3—O1—C6—C11	−46.61 (19)
C6—O1—C3—C4	134.31 (15)	C3—O1—C6—C7	136.93 (15)
F2—C2—C3—O1	1.0 (2)	C11—C6—C7—C8	−0.5 (2)
C1—C2—C3—O1	−178.94 (14)	O1—C6—C7—C8	175.90 (14)
F2—C2—C3—C4	177.58 (14)	C6—C7—C8—C9	0.1 (2)
C1—C2—C3—C4	−2.4 (2)	C7—C8—C9—C10	0.4 (2)
O1—C3—C4—F3	−1.8 (2)	C8—C9—C10—C11	−0.6 (2)
C2—C3—C4—F3	−178.48 (14)	C7—C6—C11—C10	0.3 (2)
O1—C3—C4—C5	178.73 (14)	O1—C6—C11—C10	−175.95 (14)
C2—C3—C4—C5	2.1 (2)	C9—C10—C11—C6	0.2 (2)

### 4-(4-Bromophenoxy)-2,3,5,6-tetrafluoropyridine (II) Crystal data

**Table:**

C_11_H_4_BrF_4_NO	*F*(000) = 624
*M**_r_* = 322.06	*D*_x_ = 2.004 Mg m^−^^3^
Monoclinic, *P*2_1_/*n*	Mo *K*α radiation, λ = 0.71073 Å
*a* = 13.3530 (5) Å	Cell parameters from 7652 reflections
*b* = 5.8584 (2) Å	θ = 2.6–31.9°
*c* = 14.8863 (6) Å	µ = 3.89 mm^−^^1^
β = 113.585 (2)°	*T* = 100 K
*V* = 1067.24 (7) Å^3^	Rectangular prism, colourless
*Z* = 4	0.15 × 0.10 × 0.09 mm

### 4-(4-Bromophenoxy)-2,3,5,6-tetrafluoropyridine (II) Data collection

**Table:**

Bruker SMART APEX CCD diffractometer	3544 independent reflections
Radiation source: fine focus sealed tube	3013 reflections with *I* > 2σ(*I*)
Graphite monochromator	*R*_int_ = 0.040
Detector resolution: 8.3333 pixels mm^-1^	θ_max_ = 31.5°, θ_min_ = 1.7°
ω Scans scans	*h* = −19→19
Absorption correction: multi-scan (SADABS; Bruker, 2017)	*k* = −8→8
*T*_min_ = 0.21, *T*_max_ = 0.72	*l* = −21→21
19174 measured reflections	

### 4-(4-Bromophenoxy)-2,3,5,6-tetrafluoropyridine (II) Refinement

**Table:**

Refinement on *F*^2^	Primary atom site location: structure-invariant direct methods
Least-squares matrix: full	Hydrogen site location: inferred from neighbouring sites
*R*[*F*^2^ > 2σ(*F*^2^)] = 0.031	H-atom parameters constrained
*wR*(*F*^2^) = 0.083	*w* = 1/[σ^2^(*F*_o_^2^) + (0.050*P*)^2^] where *P* = (*F*_o_^2^ + 2*F*_c_^2^)/3
*S* = 1.06	(Δ/σ)_max_ = 0.002
3544 reflections	Δρ_max_ = 0.84 e Å^−^^3^
163 parameters	Δρ_min_ = −0.77 e Å^−^^3^
0 restraints	

### 4-(4-Bromophenoxy)-2,3,5,6-tetrafluoropyridine (II) Special details

**Table:**

Geometry. All esds (except the esd in the dihedral angle between two l.s. planes) are estimated using the full covariance matrix. The cell esds are taken into account individually in the estimation of esds in distances, angles and torsion angles; correlations between esds in cell parameters are only used when they are defined by crystal symmetry. An approximate (isotropic) treatment of cell esds is used for estimating esds involving l.s. planes.

### 4-(4-Bromophenoxy)-2,3,5,6-tetrafluoropyridine (II) Fractional atomic coordinates and isotropic or equivalent isotropic displacement parameters (Å^2^)

**Table:**

	*x*	*y*	*z*	*U*_iso_*/*U*_eq_	
Br1	0.69708 (2)	0.16236 (3)	0.49318 (2)	0.01909 (7)	
F1	0.26006 (9)	0.89335 (18)	0.84433 (9)	0.0217 (2)	
F2	0.38676 (8)	1.02091 (16)	0.74927 (8)	0.0180 (2)	
F3	0.57954 (9)	0.32539 (15)	0.85557 (9)	0.0198 (2)	
F4	0.44206 (9)	0.22697 (18)	0.94178 (8)	0.0204 (2)	
O1	0.55019 (9)	0.75025 (19)	0.75137 (9)	0.0171 (3)	
N1	0.35097 (12)	0.5592 (2)	0.89200 (11)	0.0165 (3)	
C1	0.33969 (13)	0.7540 (3)	0.84459 (13)	0.0158 (3)	
C2	0.40295 (14)	0.8195 (2)	0.79609 (13)	0.0138 (3)	
C3	0.48559 (14)	0.6752 (3)	0.79564 (13)	0.0139 (3)	
C4	0.49951 (13)	0.4716 (3)	0.84799 (12)	0.0146 (3)	
C5	0.42904 (14)	0.4238 (3)	0.89247 (12)	0.0151 (3)	
C6	0.57942 (13)	0.6035 (3)	0.69117 (12)	0.0140 (3)	
C7	0.52283 (14)	0.4050 (3)	0.65069 (13)	0.0161 (3)	
H7	0.460999	0.360861	0.662945	0.019*	
C8	0.55832 (14)	0.2716 (3)	0.59177 (13)	0.0167 (3)	
H8	0.522146	0.13251	0.564996	0.02*	
C9	0.64660 (14)	0.3426 (3)	0.57230 (13)	0.0147 (3)	
C10	0.70034 (14)	0.5462 (3)	0.61029 (13)	0.0162 (3)	
H10	0.759498	0.595176	0.594964	0.019*	
C11	0.66669 (14)	0.6774 (3)	0.67088 (13)	0.0153 (3)	
H11	0.703098	0.816021	0.698056	0.018*	

### 4-(4-Bromophenoxy)-2,3,5,6-tetrafluoropyridine (II) Atomic displacement parameters (Å^2^)

**Table:**

	*U*^11^	*U*^22^	*U*^33^	*U*^12^	*U*^13^	*U*^23^
Br1	0.02336 (11)	0.01936 (10)	0.01944 (11)	0.00095 (6)	0.01371 (8)	−0.00288 (6)
F1	0.0176 (5)	0.0205 (5)	0.0311 (7)	0.0047 (4)	0.0139 (5)	−0.0027 (4)
F2	0.0211 (5)	0.0126 (4)	0.0200 (5)	0.0021 (4)	0.0079 (4)	0.0014 (4)
F3	0.0176 (5)	0.0176 (5)	0.0239 (6)	0.0066 (4)	0.0081 (4)	0.0008 (4)
F4	0.0280 (6)	0.0157 (5)	0.0170 (5)	−0.0011 (4)	0.0082 (4)	0.0035 (4)
O1	0.0209 (6)	0.0151 (6)	0.0210 (7)	−0.0036 (5)	0.0145 (5)	−0.0047 (5)
N1	0.0169 (7)	0.0177 (6)	0.0166 (7)	−0.0019 (5)	0.0086 (6)	−0.0027 (5)
C1	0.0138 (7)	0.0155 (7)	0.0182 (9)	0.0004 (6)	0.0065 (6)	−0.0041 (6)
C2	0.0158 (7)	0.0110 (7)	0.0134 (8)	−0.0011 (5)	0.0047 (6)	−0.0020 (5)
C3	0.0136 (7)	0.0155 (7)	0.0137 (8)	−0.0031 (5)	0.0064 (6)	−0.0037 (5)
C4	0.0132 (7)	0.0149 (7)	0.0143 (8)	0.0018 (6)	0.0040 (6)	−0.0020 (5)
C5	0.0196 (8)	0.0136 (7)	0.0107 (8)	−0.0006 (6)	0.0047 (6)	0.0006 (5)
C6	0.0147 (7)	0.0142 (7)	0.0142 (8)	0.0006 (6)	0.0068 (6)	−0.0016 (6)
C7	0.0151 (7)	0.0176 (7)	0.0181 (8)	−0.0044 (6)	0.0094 (6)	−0.0042 (6)
C8	0.0189 (8)	0.0156 (7)	0.0172 (9)	−0.0040 (6)	0.0091 (7)	−0.0045 (6)
C9	0.0153 (7)	0.0167 (7)	0.0132 (8)	0.0016 (6)	0.0068 (6)	−0.0003 (5)
C10	0.0143 (7)	0.0186 (7)	0.0174 (8)	−0.0022 (6)	0.0080 (6)	−0.0009 (6)
C11	0.0151 (7)	0.0166 (7)	0.0155 (8)	−0.0041 (6)	0.0076 (7)	−0.0014 (6)

### 4-(4-Bromophenoxy)-2,3,5,6-tetrafluoropyridine (II) Geometric parameters (Å, º)

**Table:**

Br1—C9	1.8952 (17)	C4—C5	1.379 (2)
F1—C1	1.3395 (19)	C6—C11	1.385 (2)
F2—C2	1.3430 (18)	C6—C7	1.386 (2)
F3—C4	1.3386 (18)	C7—C8	1.392 (2)
F4—C5	1.3401 (19)	C7—H7	0.95
O1—C3	1.352 (2)	C8—C9	1.385 (2)
O1—C6	1.4050 (19)	C8—H8	0.95
N1—C5	1.308 (2)	C9—C10	1.390 (2)
N1—C1	1.318 (2)	C10—C11	1.389 (2)
C1—C2	1.368 (2)	C10—H10	0.95
C2—C3	1.392 (2)	C11—H11	0.95
C3—C4	1.396 (2)		
			
C3—O1—C6	120.41 (13)	C11—C6—O1	114.89 (14)
C5—N1—C1	116.63 (15)	C7—C6—O1	123.25 (15)
N1—C1—F1	116.72 (15)	C6—C7—C8	118.76 (16)
N1—C1—C2	124.29 (15)	C6—C7—H7	120.6
F1—C1—C2	118.99 (16)	C8—C7—H7	120.6
F2—C2—C1	121.02 (15)	C9—C8—C7	119.74 (16)
F2—C2—C3	119.63 (15)	C9—C8—H8	120.1
C1—C2—C3	119.34 (15)	C7—C8—H8	120.1
O1—C3—C2	117.79 (14)	C8—C9—C10	121.07 (16)
O1—C3—C4	125.59 (15)	C8—C9—Br1	120.46 (12)
C2—C3—C4	116.43 (15)	C10—C9—Br1	118.47 (13)
F3—C4—C5	120.31 (15)	C11—C10—C9	119.35 (16)
F3—C4—C3	121.15 (15)	C11—C10—H10	120.3
C5—C4—C3	118.54 (15)	C9—C10—H10	120.3
N1—C5—F4	116.96 (15)	C6—C11—C10	119.20 (15)
N1—C5—C4	124.73 (16)	C6—C11—H11	120.4
F4—C5—C4	118.29 (15)	C10—C11—H11	120.4
C11—C6—C7	121.80 (16)		
			
C5—N1—C1—F1	−179.75 (15)	C1—N1—C5—C4	0.5 (3)
C5—N1—C1—C2	0.7 (3)	F3—C4—C5—N1	177.56 (15)
N1—C1—C2—F2	−179.99 (15)	C3—C4—C5—N1	−2.2 (3)
F1—C1—C2—F2	0.5 (2)	F3—C4—C5—F4	−0.9 (2)
N1—C1—C2—C3	−0.2 (3)	C3—C4—C5—F4	179.31 (14)
F1—C1—C2—C3	−179.71 (15)	C3—O1—C6—C11	−163.96 (15)
C6—O1—C3—C2	−137.39 (15)	C3—O1—C6—C7	19.0 (2)
C6—O1—C3—C4	47.9 (2)	C11—C6—C7—C8	3.0 (3)
F2—C2—C3—O1	3.2 (2)	O1—C6—C7—C8	179.83 (16)
C1—C2—C3—O1	−176.63 (15)	C6—C7—C8—C9	−2.0 (3)
F2—C2—C3—C4	178.33 (14)	C7—C8—C9—C10	−0.4 (3)
C1—C2—C3—C4	−1.5 (2)	C7—C8—C9—Br1	179.46 (13)
O1—C3—C4—F3	−2.5 (3)	C8—C9—C10—C11	1.7 (3)
C2—C3—C4—F3	−177.21 (15)	Br1—C9—C10—C11	−178.07 (13)
O1—C3—C4—C5	177.29 (16)	C7—C6—C11—C10	−1.7 (3)
C2—C3—C4—C5	2.5 (2)	O1—C6—C11—C10	−178.70 (15)
C1—N1—C5—F4	179.03 (14)	C9—C10—C11—C6	−0.8 (3)

### 2,3,5,6-Tetrafluoro-4-[(naphthalen-2-yl)oxy]pyridine (III) Crystal data

**Table:**

C_15_H_7_F_4_NO	*D*_x_ = 1.596 Mg m^−^^3^
*M**_r_* = 293.22	Mo *K*α radiation, λ = 0.71073 Å
Orthorhombic, *P*2_1_2_1_2_1_	Cell parameters from 9963 reflections
*a* = 5.4703 (5) Å	θ = 2.4–29.2°
*b* = 9.2548 (9) Å	µ = 0.14 mm^−^^1^
*c* = 24.109 (2) Å	*T* = 100 K
*V* = 1220.6 (2) Å^3^	Rectangular prism, colourless
*Z* = 4	0.54 × 0.36 × 0.29 mm
*F*(000) = 592	

### 2,3,5,6-Tetrafluoro-4-[(naphthalen-2-yl)oxy]pyridine (III) Data collection

**Table:**

Bruker SMART APEX CCD diffractometer	3296 independent reflections
Radiation source: fine focus sealed tube	3149 reflections with *I* > 2σ(*I*)
Graphite monochromator	*R*_int_ = 0.025
Detector resolution: 8.3333 pixels mm^-1^	θ_max_ = 29.1°, θ_min_ = 2.4°
ω Scans scans	*h* = −7→7
Absorption correction: multi-scan (SADABS; Bruker, 2017)	*k* = −12→12
*T*_min_ = 0.83, *T*_max_ = 0.96	*l* = −32→33
26820 measured reflections	

### 2,3,5,6-Tetrafluoro-4-[(naphthalen-2-yl)oxy]pyridine (III) Refinement

**Table:**

Refinement on *F*^2^	Hydrogen site location: inferred from neighbouring sites
Least-squares matrix: full	H-atom parameters constrained
*R*[*F*^2^ > 2σ(*F*^2^)] = 0.031	*w* = 1/[σ^2^(*F*_o_^2^) + (0.0385*P*)^2^ + 0.2387*P*] where *P* = (*F*_o_^2^ + 2*F*_c_^2^)/3
*wR*(*F*^2^) = 0.078	(Δ/σ)_max_ < 0.001
*S* = 1.07	Δρ_max_ = 0.27 e Å^−^^3^
3296 reflections	Δρ_min_ = −0.15 e Å^−^^3^
190 parameters	Absolute structure: Flack *x* determined using 1255 quotients [(*I*^+^)-(*I*^-^)]/[(*I*^+^)+(*I*^-^)] (Parsons *et al.*, 2013)
0 restraints	Absolute structure parameter: −0.08 (14)
Primary atom site location: structure-invariant direct methods	

### 2,3,5,6-Tetrafluoro-4-[(naphthalen-2-yl)oxy]pyridine (III) Special details

**Table:**

Geometry. All esds (except the esd in the dihedral angle between two l.s. planes) are estimated using the full covariance matrix. The cell esds are taken into account individually in the estimation of esds in distances, angles and torsion angles; correlations between esds in cell parameters are only used when they are defined by crystal symmetry. An approximate (isotropic) treatment of cell esds is used for estimating esds involving l.s. planes.

### 2,3,5,6-Tetrafluoro-4-[(naphthalen-2-yl)oxy]pyridine (III) Fractional atomic coordinates and isotropic or equivalent isotropic displacement parameters (Å^2^)

**Table:**

	*x*	*y*	*z*	*U*_iso_*/*U*_eq_	
F1	0.1901 (3)	0.27940 (14)	0.13034 (5)	0.0479 (3)	
F2	0.5809 (2)	0.39295 (12)	0.18393 (4)	0.0389 (3)	
F3	0.2850 (2)	0.16943 (11)	0.34729 (4)	0.0312 (2)	
F4	−0.0860 (2)	0.06445 (13)	0.28603 (6)	0.0493 (3)	
O1	0.6420 (2)	0.34634 (14)	0.29382 (5)	0.0277 (3)	
N1	0.0540 (3)	0.17233 (17)	0.20838 (7)	0.0356 (3)	
C1	0.2194 (4)	0.25229 (19)	0.18427 (7)	0.0320 (4)	
C2	0.4193 (3)	0.31157 (17)	0.21119 (6)	0.0265 (3)	
C3	0.4454 (3)	0.28847 (16)	0.26793 (6)	0.0222 (3)	
C4	0.2711 (3)	0.20160 (17)	0.29324 (6)	0.0246 (3)	
C5	0.0823 (3)	0.14819 (18)	0.26127 (8)	0.0320 (4)	
C6	0.6094 (3)	0.40005 (17)	0.34853 (6)	0.0228 (3)	
C7	0.7792 (3)	0.36372 (17)	0.38711 (6)	0.0241 (3)	
H7	0.908206	0.299232	0.377994	0.029*	
C8	0.7619 (3)	0.42322 (17)	0.44129 (6)	0.0226 (3)	
C9	0.9344 (3)	0.3885 (2)	0.48337 (7)	0.0285 (3)	
H9	1.064542	0.323683	0.475557	0.034*	
C10	0.9133 (3)	0.4482 (2)	0.53513 (7)	0.0320 (4)	
H10	1.030211	0.425143	0.562892	0.038*	
C11	0.7196 (4)	0.5437 (2)	0.54765 (7)	0.0326 (4)	
H11	0.707125	0.584129	0.583766	0.039*	
C12	0.5492 (4)	0.57864 (19)	0.50802 (7)	0.0294 (3)	
H12	0.419415	0.642863	0.516865	0.035*	
C13	0.5662 (3)	0.51895 (17)	0.45372 (6)	0.0233 (3)	
C14	0.3926 (3)	0.55230 (17)	0.41174 (7)	0.0250 (3)	
H14	0.260636	0.615515	0.419906	0.03*	
C15	0.4135 (3)	0.49422 (17)	0.35952 (6)	0.0242 (3)	
H15	0.298037	0.51716	0.331426	0.029*	

### 2,3,5,6-Tetrafluoro-4-[(naphthalen-2-yl)oxy]pyridine (III) Atomic displacement parameters (Å^2^)

**Table:**

	*U*^11^	*U*^22^	*U*^33^	*U*^12^	*U*^13^	*U*^23^
F1	0.0678 (8)	0.0484 (7)	0.0276 (5)	0.0196 (6)	−0.0202 (6)	−0.0095 (5)
F2	0.0582 (7)	0.0356 (5)	0.0229 (4)	−0.0087 (5)	0.0076 (5)	0.0027 (4)
F3	0.0379 (6)	0.0285 (5)	0.0271 (5)	−0.0018 (4)	0.0053 (4)	0.0050 (4)
F4	0.0365 (6)	0.0367 (6)	0.0748 (9)	−0.0157 (5)	0.0055 (6)	−0.0067 (6)
O1	0.0237 (5)	0.0394 (6)	0.0199 (5)	−0.0060 (5)	0.0015 (4)	−0.0052 (5)
N1	0.0318 (7)	0.0300 (7)	0.0449 (8)	0.0049 (6)	−0.0128 (7)	−0.0146 (6)
C1	0.0416 (10)	0.0272 (8)	0.0271 (8)	0.0118 (7)	−0.0106 (7)	−0.0084 (6)
C2	0.0358 (8)	0.0221 (7)	0.0216 (7)	0.0024 (7)	−0.0004 (6)	−0.0010 (5)
C3	0.0234 (7)	0.0210 (6)	0.0221 (7)	0.0008 (6)	−0.0015 (6)	−0.0011 (5)
C4	0.0267 (7)	0.0212 (6)	0.0260 (7)	0.0015 (6)	0.0005 (6)	0.0001 (6)
C5	0.0261 (8)	0.0224 (7)	0.0474 (10)	−0.0016 (6)	−0.0013 (7)	−0.0064 (7)
C6	0.0228 (7)	0.0273 (7)	0.0182 (6)	−0.0047 (6)	0.0010 (5)	−0.0003 (6)
C7	0.0192 (6)	0.0293 (7)	0.0237 (6)	−0.0001 (6)	0.0012 (5)	−0.0001 (6)
C8	0.0200 (7)	0.0264 (7)	0.0215 (6)	−0.0026 (6)	−0.0010 (6)	0.0033 (5)
C9	0.0230 (7)	0.0378 (9)	0.0248 (7)	0.0001 (7)	−0.0019 (6)	0.0061 (6)
C10	0.0306 (8)	0.0429 (10)	0.0227 (7)	−0.0051 (8)	−0.0055 (7)	0.0062 (7)
C11	0.0396 (9)	0.0379 (9)	0.0204 (7)	−0.0058 (8)	−0.0011 (7)	−0.0016 (6)
C12	0.0333 (8)	0.0298 (8)	0.0251 (7)	−0.0003 (7)	0.0009 (7)	−0.0028 (6)
C13	0.0246 (7)	0.0231 (7)	0.0222 (7)	−0.0034 (6)	−0.0001 (6)	0.0015 (5)
C14	0.0250 (7)	0.0234 (7)	0.0264 (7)	0.0018 (6)	−0.0010 (6)	0.0003 (6)
C15	0.0237 (7)	0.0259 (7)	0.0231 (7)	−0.0016 (6)	−0.0041 (6)	0.0025 (5)

### 2,3,5,6-Tetrafluoro-4-[(naphthalen-2-yl)oxy]pyridine (III) Geometric parameters (Å, º)

**Table:**

F1—C1	1.334 (2)	C7—H7	0.95
F2—C2	1.334 (2)	C8—C13	1.421 (2)
F3—C4	1.3389 (18)	C8—C9	1.422 (2)
F4—C5	1.343 (2)	C9—C10	1.369 (2)
O1—C3	1.3538 (19)	C9—H9	0.95
O1—C6	1.4209 (18)	C10—C11	1.413 (3)
N1—C5	1.304 (3)	C10—H10	0.95
N1—C1	1.305 (3)	C11—C12	1.374 (2)
C1—C2	1.385 (3)	C11—H11	0.95
C2—C3	1.392 (2)	C12—C13	1.424 (2)
C3—C4	1.389 (2)	C12—H12	0.95
C4—C5	1.380 (2)	C13—C14	1.421 (2)
C6—C7	1.357 (2)	C14—C15	1.374 (2)
C6—C15	1.407 (2)	C14—H14	0.95
C7—C8	1.421 (2)	C15—H15	0.95
			
C3—O1—C6	117.82 (12)	C7—C8—C9	121.61 (15)
C5—N1—C1	116.78 (16)	C13—C8—C9	119.34 (14)
N1—C1—F1	117.23 (17)	C10—C9—C8	120.20 (16)
N1—C1—C2	124.26 (16)	C10—C9—H9	119.9
F1—C1—C2	118.50 (18)	C8—C9—H9	119.9
F2—C2—C1	121.05 (15)	C9—C10—C11	120.68 (15)
F2—C2—C3	120.19 (16)	C9—C10—H10	119.7
C1—C2—C3	118.73 (16)	C11—C10—H10	119.7
O1—C3—C4	124.90 (14)	C12—C11—C10	120.51 (15)
O1—C3—C2	118.27 (15)	C12—C11—H11	119.7
C4—C3—C2	116.76 (15)	C10—C11—H11	119.7
F3—C4—C5	120.43 (15)	C11—C12—C13	120.26 (16)
F3—C4—C3	121.15 (14)	C11—C12—H12	119.9
C5—C4—C3	118.42 (15)	C13—C12—H12	119.9
N1—C5—F4	116.90 (17)	C14—C13—C8	119.22 (13)
N1—C5—C4	125.00 (17)	C14—C13—C12	121.77 (15)
F4—C5—C4	118.11 (17)	C8—C13—C12	119.00 (14)
C7—C6—C15	123.10 (14)	C15—C14—C13	120.81 (15)
C7—C6—O1	117.64 (14)	C15—C14—H14	119.6
C15—C6—O1	119.15 (13)	C13—C14—H14	119.6
C6—C7—C8	119.25 (14)	C14—C15—C6	118.57 (14)
C6—C7—H7	120.4	C14—C15—H15	120.7
C8—C7—H7	120.4	C6—C15—H15	120.7
C7—C8—C13	119.05 (13)		
			
C5—N1—C1—F1	−179.02 (15)	C3—O1—C6—C7	134.10 (15)
C5—N1—C1—C2	0.1 (3)	C3—O1—C6—C15	−49.6 (2)
N1—C1—C2—F2	−179.70 (15)	C15—C6—C7—C8	−0.4 (2)
F1—C1—C2—F2	−0.6 (2)	O1—C6—C7—C8	175.80 (13)
N1—C1—C2—C3	−1.6 (3)	C6—C7—C8—C13	0.3 (2)
F1—C1—C2—C3	177.43 (14)	C6—C7—C8—C9	179.80 (15)
C6—O1—C3—C4	−39.6 (2)	C7—C8—C9—C10	179.68 (15)
C6—O1—C3—C2	143.33 (15)	C13—C8—C9—C10	−0.8 (2)
F2—C2—C3—O1	−2.2 (2)	C8—C9—C10—C11	0.6 (3)
C1—C2—C3—O1	179.72 (14)	C9—C10—C11—C12	−0.2 (3)
F2—C2—C3—C4	−179.48 (15)	C10—C11—C12—C13	−0.1 (3)
C1—C2—C3—C4	2.4 (2)	C7—C8—C13—C14	0.2 (2)
O1—C3—C4—F3	0.8 (2)	C9—C8—C13—C14	−179.35 (15)
C2—C3—C4—F3	177.89 (14)	C7—C8—C13—C12	−179.93 (15)
O1—C3—C4—C5	−178.91 (15)	C9—C8—C13—C12	0.5 (2)
C2—C3—C4—C5	−1.8 (2)	C11—C12—C13—C14	179.78 (16)
C1—N1—C5—F4	−179.19 (15)	C11—C12—C13—C8	−0.1 (2)
C1—N1—C5—C4	0.6 (3)	C8—C13—C14—C15	−0.6 (2)
F3—C4—C5—N1	−179.41 (16)	C12—C13—C14—C15	179.57 (16)
C3—C4—C5—N1	0.3 (3)	C13—C14—C15—C6	0.5 (2)
F3—C4—C5—F4	0.4 (2)	C7—C6—C15—C14	0.0 (2)
C3—C4—C5—F4	−179.88 (14)	O1—C6—C15—C14	−176.10 (14)

### 4-[(6-Bromonaphthalen-2-yl)oxy]-2,3,5,6-tetrafluoropyridine (IV) Crystal data

**Table:**

C_15_H_6_BrF_4_NO	*F*(000) = 364
*M**_r_* = 372.12	*D*_x_ = 1.911 Mg m^−^^3^
Monoclinic, *P*2_1_	Mo *K*α radiation, λ = 0.71073 Å
*a* = 6.0135 (3) Å	Cell parameters from 7392 reflections
*b* = 7.4994 (4) Å	θ = 3.1–27.4°
*c* = 14.6318 (7) Å	µ = 3.23 mm^−^^1^
β = 101.401 (2)°	*T* = 100 K
*V* = 646.84 (6) Å^3^	Plate, colourless
*Z* = 2	0.30 × 0.14 × 0.04 mm

### 4-[(6-Bromonaphthalen-2-yl)oxy]-2,3,5,6-tetrafluoropyridine (IV) Data collection

**Table:**

Bruker SMART APEX CCD diffractometer	2775 independent reflections
Radiation source: fine focus sealed tube	2566 reflections with *I* > 2σ(*I*)
Graphite monochromator	*R*_int_ = 0.040
Detector resolution: 8.3333 pixels mm^-1^	θ_max_ = 27.1°, θ_min_ = 1.4°
ω Scans scans	*h* = −7→7
Absorption correction: multi-scan (SADABS; Bruker, 2017)	*k* = −9→9
*T*_min_ = 0.63, *T*_max_ = 0.89	*l* = −18→18
12954 measured reflections	

### 4-[(6-Bromonaphthalen-2-yl)oxy]-2,3,5,6-tetrafluoropyridine (IV) Refinement

**Table:**

Refinement on *F*^2^	Hydrogen site location: inferred from neighbouring sites
Least-squares matrix: full	H-atom parameters constrained
*R*[*F*^2^ > 2σ(*F*^2^)] = 0.031	*w* = 1/[σ^2^(*F*_o_^2^) + (0.0174*P*)^2^ + 0.147*P*] where *P* = (*F*_o_^2^ + 2*F*_c_^2^)/3
*wR*(*F*^2^) = 0.058	(Δ/σ)_max_ = 0.001
*S* = 1.14	Δρ_max_ = 0.70 e Å^−^^3^
2775 reflections	Δρ_min_ = −0.46 e Å^−^^3^
200 parameters	Absolute structure: Refined as an inversion twin
1 restraint	Absolute structure parameter: 0.171 (12)
Primary atom site location: structure-invariant direct methods	

### 4-[(6-Bromonaphthalen-2-yl)oxy]-2,3,5,6-tetrafluoropyridine (IV) Special details

**Table:**

Geometry. All esds (except the esd in the dihedral angle between two l.s. planes) are estimated using the full covariance matrix. The cell esds are taken into account individually in the estimation of esds in distances, angles and torsion angles; correlations between esds in cell parameters are only used when they are defined by crystal symmetry. An approximate (isotropic) treatment of cell esds is used for estimating esds involving l.s. planes.
Refinement. Refined as a 2-component inversion twin.

### 4-[(6-Bromonaphthalen-2-yl)oxy]-2,3,5,6-tetrafluoropyridine (IV) Fractional atomic coordinates and isotropic or equivalent isotropic displacement parameters (Å^2^)

**Table:**

	*x*	*y*	*z*	*U*_iso_*/*U*_eq_	
Br1	1.11387 (6)	0.12723 (7)	0.65331 (3)	0.02027 (12)	
F1	0.1437 (5)	0.8959 (4)	0.1693 (2)	0.0341 (8)	
F2	0.4300 (3)	0.6186 (6)	0.20499 (14)	0.0245 (5)	
F3	−0.1671 (5)	0.2537 (4)	0.04981 (19)	0.0325 (7)	
F4	−0.4284 (4)	0.5505 (4)	0.0217 (2)	0.0430 (9)	
O1	0.2637 (6)	0.2724 (4)	0.1423 (2)	0.0258 (8)	
N1	−0.1424 (7)	0.7231 (7)	0.0963 (3)	0.0276 (11)	
C1	0.0692 (8)	0.7344 (7)	0.1415 (3)	0.0242 (11)	
C2	0.2157 (7)	0.5921 (6)	0.1599 (3)	0.0163 (12)	
C3	0.1384 (8)	0.4227 (8)	0.1316 (3)	0.0187 (11)	
C4	−0.0837 (8)	0.4126 (7)	0.0826 (3)	0.0227 (11)	
C5	−0.2127 (9)	0.5642 (8)	0.0680 (4)	0.0277 (15)	
C6	0.4160 (7)	0.2377 (6)	0.2262 (3)	0.0196 (10)	
C7	0.3753 (7)	0.2843 (6)	0.3113 (3)	0.0186 (10)	
H7	0.238946	0.343679	0.31662	0.022*	
C8	0.5417 (7)	0.2423 (5)	0.3922 (3)	0.0148 (9)	
C9	0.5119 (7)	0.2886 (6)	0.4829 (3)	0.0164 (9)	
H9	0.375158	0.344584	0.490712	0.02*	
C10	0.6783 (7)	0.2534 (6)	0.5599 (3)	0.0165 (9)	
H10	0.657322	0.284878	0.620469	0.02*	
C11	0.8803 (7)	0.1702 (5)	0.5475 (3)	0.0157 (11)	
C12	0.9146 (6)	0.1211 (10)	0.4619 (2)	0.0165 (7)	
H12	1.052023	0.064184	0.45575	0.02*	
C13	0.7438 (6)	0.1551 (7)	0.3814 (3)	0.0150 (11)	
C14	0.7724 (7)	0.1040 (8)	0.2918 (3)	0.0199 (11)	
H14	0.90638	0.042866	0.284841	0.024*	
C15	0.6108 (6)	0.1412 (10)	0.2154 (3)	0.0208 (9)	
H15	0.628527	0.10273	0.155347	0.025*	

### 4-[(6-Bromonaphthalen-2-yl)oxy]-2,3,5,6-tetrafluoropyridine (IV) Atomic displacement parameters (Å^2^)

**Table:**

	*U*^11^	*U*^22^	*U*^33^	*U*^12^	*U*^13^	*U*^23^
Br1	0.01749 (18)	0.0207 (2)	0.0211 (2)	0.0017 (3)	0.00019 (13)	0.0006 (3)
F1	0.047 (2)	0.021 (2)	0.036 (2)	0.0032 (15)	0.0129 (16)	0.0016 (15)
F2	0.0190 (10)	0.0269 (13)	0.0241 (12)	−0.0047 (19)	−0.0044 (9)	−0.003 (2)
F3	0.0324 (15)	0.0414 (19)	0.0232 (15)	−0.0209 (14)	0.0041 (12)	−0.0085 (13)
F4	0.0148 (13)	0.082 (3)	0.0296 (16)	0.0036 (13)	−0.0019 (12)	0.0034 (15)
O1	0.037 (2)	0.0213 (19)	0.0158 (17)	0.0043 (15)	−0.0035 (14)	−0.0020 (14)
N1	0.022 (3)	0.039 (3)	0.022 (3)	0.010 (2)	0.005 (2)	0.007 (2)
C1	0.036 (3)	0.020 (3)	0.019 (3)	0.004 (2)	0.011 (2)	0.001 (2)
C2	0.0181 (19)	0.018 (4)	0.0127 (19)	−0.0040 (19)	0.0015 (15)	0.0025 (18)
C3	0.022 (3)	0.023 (3)	0.011 (2)	−0.001 (2)	0.0038 (19)	0.004 (2)
C4	0.022 (2)	0.035 (3)	0.012 (2)	−0.010 (2)	0.0045 (18)	−0.002 (2)
C5	0.015 (3)	0.051 (4)	0.017 (3)	0.001 (2)	0.001 (2)	0.004 (2)
C6	0.026 (2)	0.014 (2)	0.017 (2)	−0.0022 (19)	−0.0007 (18)	−0.0005 (19)
C7	0.018 (2)	0.016 (2)	0.021 (2)	0.0012 (17)	0.0039 (18)	0.0000 (19)
C8	0.018 (2)	0.008 (2)	0.019 (2)	−0.0034 (16)	0.0052 (17)	−0.0006 (17)
C9	0.016 (2)	0.013 (2)	0.021 (2)	0.0029 (17)	0.0047 (18)	−0.0009 (18)
C10	0.019 (2)	0.014 (2)	0.017 (2)	−0.0024 (18)	0.0045 (18)	−0.0025 (18)
C11	0.0161 (19)	0.009 (3)	0.021 (2)	−0.0012 (15)	0.0009 (16)	0.0038 (16)
C12	0.0174 (17)	0.0099 (18)	0.0233 (19)	0.000 (3)	0.0069 (14)	0.000 (3)
C13	0.0178 (18)	0.009 (3)	0.020 (2)	−0.0012 (17)	0.0077 (15)	0.0013 (19)
C14	0.0220 (19)	0.016 (3)	0.024 (2)	0.000 (2)	0.0118 (16)	−0.004 (2)
C15	0.028 (2)	0.020 (3)	0.0167 (19)	−0.003 (3)	0.0091 (15)	0.001 (3)

### 4-[(6-Bromonaphthalen-2-yl)oxy]-2,3,5,6-tetrafluoropyridine (IV) Geometric parameters (Å, º)

**Table:**

Br1—C11	1.901 (4)	C7—C8	1.427 (6)
F1—C1	1.328 (6)	C7—H7	0.95
F2—C2	1.342 (4)	C8—C13	1.416 (6)
F3—C4	1.344 (6)	C8—C9	1.417 (6)
F4—C5	1.344 (6)	C9—C10	1.376 (6)
O1—C3	1.348 (6)	C9—H9	0.95
O1—C6	1.403 (5)	C10—C11	1.408 (6)
N1—C5	1.304 (7)	C10—H10	0.95
N1—C1	1.316 (6)	C11—C12	1.360 (6)
C1—C2	1.376 (6)	C12—C13	1.424 (5)
C2—C3	1.387 (7)	C12—H12	0.95
C3—C4	1.388 (6)	C13—C14	1.408 (6)
C4—C5	1.369 (7)	C14—C15	1.358 (6)
C6—C7	1.361 (6)	C14—H14	0.95
C6—C15	1.412 (7)	C15—H15	0.95
			
C3—O1—C6	120.7 (4)	C13—C8—C7	119.1 (4)
C5—N1—C1	116.0 (5)	C9—C8—C7	121.7 (4)
N1—C1—F1	116.6 (5)	C10—C9—C8	120.9 (4)
N1—C1—C2	124.5 (5)	C10—C9—H9	119.6
F1—C1—C2	118.9 (4)	C8—C9—H9	119.6
F2—C2—C1	119.8 (4)	C9—C10—C11	119.1 (4)
F2—C2—C3	121.0 (4)	C9—C10—H10	120.5
C1—C2—C3	119.2 (5)	C11—C10—H10	120.5
O1—C3—C2	125.8 (4)	C12—C11—C10	122.0 (4)
O1—C3—C4	118.2 (5)	C12—C11—Br1	118.8 (3)
C2—C3—C4	115.8 (5)	C10—C11—Br1	119.2 (3)
F3—C4—C5	121.4 (4)	C11—C12—C13	119.8 (4)
F3—C4—C3	119.1 (5)	C11—C12—H12	120.1
C5—C4—C3	119.5 (5)	C13—C12—H12	120.1
N1—C5—F4	116.7 (4)	C14—C13—C8	119.7 (4)
N1—C5—C4	124.8 (5)	C14—C13—C12	121.3 (4)
F4—C5—C4	118.4 (5)	C8—C13—C12	119.0 (4)
C7—C6—O1	123.3 (4)	C15—C14—C13	120.8 (4)
C7—C6—C15	122.5 (4)	C15—C14—H14	119.6
O1—C6—C15	114.2 (4)	C13—C14—H14	119.6
C6—C7—C8	118.7 (4)	C14—C15—C6	119.2 (4)
C6—C7—H7	120.7	C14—C15—H15	120.4
C8—C7—H7	120.7	C6—C15—H15	120.4
C13—C8—C9	119.2 (4)		
			
C5—N1—C1—F1	179.0 (5)	C3—O1—C6—C15	−147.8 (5)
C5—N1—C1—C2	0.0 (8)	O1—C6—C7—C8	179.9 (4)
N1—C1—C2—F2	178.6 (4)	C15—C6—C7—C8	3.4 (7)
F1—C1—C2—F2	−0.3 (6)	C6—C7—C8—C13	0.5 (6)
N1—C1—C2—C3	−1.5 (7)	C6—C7—C8—C9	179.3 (4)
F1—C1—C2—C3	179.5 (4)	C13—C8—C9—C10	1.5 (6)
C6—O1—C3—C2	42.9 (7)	C7—C8—C9—C10	−177.3 (4)
C6—O1—C3—C4	−141.8 (4)	C8—C9—C10—C11	0.0 (6)
F2—C2—C3—O1	−2.3 (7)	C9—C10—C11—C12	−1.0 (7)
C1—C2—C3—O1	177.8 (4)	C9—C10—C11—Br1	178.3 (3)
F2—C2—C3—C4	−177.7 (4)	C10—C11—C12—C13	0.6 (8)
C1—C2—C3—C4	2.4 (7)	Br1—C11—C12—C13	−178.8 (4)
O1—C3—C4—F3	1.6 (7)	C9—C8—C13—C14	178.2 (5)
C2—C3—C4—F3	177.4 (4)	C7—C8—C13—C14	−3.0 (7)
O1—C3—C4—C5	−177.8 (4)	C9—C8—C13—C12	−1.9 (7)
C2—C3—C4—C5	−2.0 (7)	C7—C8—C13—C12	176.9 (4)
C1—N1—C5—F4	180.0 (4)	C11—C12—C13—C14	−179.2 (5)
C1—N1—C5—C4	0.5 (9)	C11—C12—C13—C8	0.9 (8)
F3—C4—C5—N1	−178.8 (5)	C8—C13—C14—C15	1.6 (8)
C3—C4—C5—N1	0.6 (9)	C12—C13—C14—C15	−178.3 (6)
F3—C4—C5—F4	1.7 (7)	C13—C14—C15—C6	2.2 (9)
C3—C4—C5—F4	−178.9 (4)	C7—C6—C15—C14	−4.9 (9)
C3—O1—C6—C7	35.4 (6)	O1—C6—C15—C14	178.4 (5)

### 2,2'-Bis[(2,3,5,6-tetrafluoropyridin-4-yl)oxy]-1,1'-biphenyl (V) Crystal data

**Table:**

C_22_H_8_F_8_N_2_O_2_	*D*_x_ = 1.738 Mg m^−^^3^
*M**_r_* = 484.30	Mo *K*α radiation, λ = 0.71073 Å
Orthorhombic, *P**b**c**n*	Cell parameters from 9894 reflections
*a* = 18.8516 (6) Å	θ = 3.1–31.0°
*b* = 10.6512 (3) Å	µ = 0.17 mm^−^^1^
*c* = 9.2196 (3) Å	*T* = 100 K
*V* = 1851.22 (10) Å^3^	Rectangular prism, colourless
*Z* = 4	0.33 × 0.27 × 0.26 mm
*F*(000) = 968	

### 2,2'-Bis[(2,3,5,6-tetrafluoropyridin-4-yl)oxy]-1,1'-biphenyl (V) Data collection

**Table:**

Bruker SMART APEX CCD diffractometer	2830 independent reflections
Radiation source: fine focus sealed tube	2554 reflections with *I* > 2σ(*I*)
Graphite monochromator	*R*_int_ = 0.028
Detector resolution: 8.3333 pixels mm^-1^	θ_max_ = 30.5°, θ_min_ = 2.2°
ω Scans scans	*h* = −26→26
Absorption correction: multi-scan (SADABS; Bruker, 2017)	*k* = −15→15
*T*_min_ = 0.87, *T*_max_ = 0.96	*l* = −13→13
36854 measured reflections	

### 2,2'-Bis[(2,3,5,6-tetrafluoropyridin-4-yl)oxy]-1,1'-biphenyl (V) Refinement

**Table:**

Refinement on *F*^2^	Primary atom site location: structure-invariant direct methods
Least-squares matrix: full	Hydrogen site location: inferred from neighbouring sites
*R*[*F*^2^ > 2σ(*F*^2^)] = 0.036	H-atom parameters constrained
*wR*(*F*^2^) = 0.098	*w* = 1/[σ^2^(*F*_o_^2^) + (0.0477*P*)^2^ + 1.0353*P*] where *P* = (*F*_o_^2^ + 2*F*_c_^2^)/3
*S* = 1.06	(Δ/σ)_max_ < 0.001
2830 reflections	Δρ_max_ = 0.54 e Å^−^^3^
154 parameters	Δρ_min_ = −0.24 e Å^−^^3^
0 restraints	

### 2,2'-Bis[(2,3,5,6-tetrafluoropyridin-4-yl)oxy]-1,1'-biphenyl (V) Special details

**Table:**

Geometry. All esds (except the esd in the dihedral angle between two l.s. planes) are estimated using the full covariance matrix. The cell esds are taken into account individually in the estimation of esds in distances, angles and torsion angles; correlations between esds in cell parameters are only used when they are defined by crystal symmetry. An approximate (isotropic) treatment of cell esds is used for estimating esds involving l.s. planes.

### 2,2'-Bis[(2,3,5,6-tetrafluoropyridin-4-yl)oxy]-1,1'-biphenyl (V) Fractional atomic coordinates and isotropic or equivalent isotropic displacement parameters (Å^2^)

**Table:**

	*x*	*y*	*z*	*U*_iso_*/*U*_eq_	
F1	0.23289 (4)	0.82100 (7)	0.58850 (9)	0.02742 (18)	
F2	0.36863 (4)	0.80572 (6)	0.50165 (8)	0.02166 (16)	
F3	0.39175 (4)	0.45355 (6)	0.81381 (8)	0.02052 (15)	
F4	0.25208 (4)	0.47778 (7)	0.87330 (8)	0.02652 (17)	
O1	0.45506 (4)	0.60742 (7)	0.62192 (8)	0.01451 (15)	
N1	0.24272 (5)	0.64972 (10)	0.73110 (11)	0.0206 (2)	
C1	0.27334 (6)	0.72890 (11)	0.64163 (12)	0.0190 (2)	
C2	0.34353 (5)	0.72303 (10)	0.59814 (11)	0.01551 (19)	
C3	0.38644 (5)	0.62907 (9)	0.65624 (11)	0.01334 (18)	
C4	0.35408 (5)	0.54608 (9)	0.75268 (11)	0.01526 (19)	
C5	0.28294 (6)	0.56017 (11)	0.78372 (12)	0.0184 (2)	
C6	0.49905 (5)	0.70787 (9)	0.57843 (11)	0.01314 (18)	
C7	0.51479 (5)	0.80445 (9)	0.67541 (11)	0.01353 (19)	
C8	0.56072 (6)	0.89900 (10)	0.62696 (11)	0.0168 (2)	
H8	0.571595	0.967538	0.689043	0.02*	
C9	0.59065 (6)	0.89380 (10)	0.48901 (12)	0.0184 (2)	
H9	0.621893	0.958404	0.457927	0.022*	
C10	0.57499 (6)	0.79449 (11)	0.39657 (12)	0.0176 (2)	
H10	0.59608	0.790723	0.30309	0.021*	
C11	0.52840 (5)	0.70047 (10)	0.44090 (11)	0.01560 (19)	
H11	0.516981	0.632684	0.378125	0.019*	

### 2,2'-Bis[(2,3,5,6-tetrafluoropyridin-4-yl)oxy]-1,1'-biphenyl (V) Atomic displacement parameters (Å^2^)

**Table:**

	*U*^11^	*U*^22^	*U*^33^	*U*^12^	*U*^13^	*U*^23^
F1	0.0200 (3)	0.0288 (4)	0.0334 (4)	0.0094 (3)	−0.0009 (3)	0.0074 (3)
F2	0.0203 (3)	0.0201 (3)	0.0246 (3)	−0.0002 (2)	0.0006 (3)	0.0094 (3)
F3	0.0197 (3)	0.0198 (3)	0.0220 (3)	0.0033 (2)	0.0021 (2)	0.0073 (3)
F4	0.0211 (3)	0.0304 (4)	0.0280 (4)	−0.0027 (3)	0.0089 (3)	0.0090 (3)
O1	0.0122 (3)	0.0132 (3)	0.0181 (3)	−0.0012 (2)	0.0020 (3)	0.0001 (3)
N1	0.0146 (4)	0.0256 (5)	0.0217 (4)	0.0017 (3)	0.0019 (3)	0.0007 (4)
C1	0.0160 (4)	0.0206 (5)	0.0203 (5)	0.0038 (4)	−0.0018 (4)	0.0006 (4)
C2	0.0156 (4)	0.0152 (4)	0.0157 (4)	−0.0001 (3)	−0.0008 (3)	0.0016 (3)
C3	0.0129 (4)	0.0141 (4)	0.0130 (4)	−0.0007 (3)	0.0000 (3)	−0.0016 (3)
C4	0.0151 (4)	0.0155 (4)	0.0152 (4)	0.0006 (3)	0.0003 (3)	0.0012 (4)
C5	0.0160 (4)	0.0217 (5)	0.0174 (5)	−0.0025 (4)	0.0030 (4)	0.0018 (4)
C6	0.0120 (4)	0.0126 (4)	0.0148 (4)	−0.0011 (3)	0.0001 (3)	0.0010 (3)
C7	0.0141 (4)	0.0135 (4)	0.0130 (4)	0.0008 (3)	−0.0003 (3)	0.0008 (3)
C8	0.0188 (5)	0.0145 (4)	0.0170 (5)	−0.0029 (4)	−0.0011 (4)	0.0002 (3)
C9	0.0185 (5)	0.0173 (5)	0.0196 (5)	−0.0037 (4)	0.0009 (4)	0.0030 (4)
C10	0.0163 (4)	0.0210 (5)	0.0153 (4)	−0.0015 (4)	0.0020 (3)	0.0018 (4)
C11	0.0153 (4)	0.0172 (4)	0.0143 (4)	−0.0008 (3)	0.0001 (3)	−0.0014 (3)

### 2,2'-Bis[(2,3,5,6-tetrafluoropyridin-4-yl)oxy]-1,1'-biphenyl (V) Geometric parameters (Å, º)

**Table:**

F1—C1	1.3356 (13)	C6—C11	1.3856 (14)
F2—C2	1.3383 (12)	C6—C7	1.3949 (14)
F3—C4	1.3390 (12)	C7—C8	1.4012 (14)
F4—C5	1.3382 (12)	C7—C7^i^	1.4842 (19)
O1—C3	1.3517 (12)	C8—C9	1.3925 (15)
O1—C6	1.4118 (12)	C8—H8	0.95
N1—C5	1.3114 (14)	C9—C10	1.3900 (15)
N1—C1	1.3134 (15)	C9—H9	0.95
C1—C2	1.3840 (15)	C10—C11	1.3934 (14)
C2—C3	1.3938 (14)	C10—H10	0.95
C3—C4	1.3942 (14)	C11—H11	0.95
C4—C5	1.3795 (14)		
			
C3—O1—C6	119.96 (8)	C11—C6—O1	116.82 (9)
C5—N1—C1	116.44 (10)	C7—C6—O1	120.12 (9)
N1—C1—F1	116.81 (10)	C6—C7—C8	117.20 (9)
N1—C1—C2	124.97 (10)	C6—C7—C7^i^	120.93 (7)
F1—C1—C2	118.22 (10)	C8—C7—C7^i^	121.85 (8)
F2—C2—C1	120.06 (9)	C9—C8—C7	120.86 (10)
F2—C2—C3	121.52 (9)	C9—C8—H8	119.6
C1—C2—C3	118.42 (10)	C7—C8—H8	119.6
O1—C3—C2	126.01 (9)	C10—C9—C8	120.28 (10)
O1—C3—C4	117.37 (9)	C10—C9—H9	119.9
C2—C3—C4	116.52 (9)	C8—C9—H9	119.9
F3—C4—C5	120.55 (9)	C9—C10—C11	120.06 (10)
F3—C4—C3	120.20 (9)	C9—C10—H10	120.0
C5—C4—C3	119.25 (10)	C11—C10—H10	120.0
N1—C5—F4	117.00 (9)	C6—C11—C10	118.63 (10)
N1—C5—C4	124.36 (10)	C6—C11—H11	120.7
F4—C5—C4	118.64 (10)	C10—C11—H11	120.7
C11—C6—C7	122.93 (9)		
			
C5—N1—C1—F1	−179.46 (10)	F3—C4—C5—N1	178.43 (10)
C5—N1—C1—C2	1.11 (17)	C3—C4—C5—N1	−1.84 (17)
N1—C1—C2—F2	177.24 (10)	F3—C4—C5—F4	−1.58 (16)
F1—C1—C2—F2	−2.18 (16)	C3—C4—C5—F4	178.15 (9)
N1—C1—C2—C3	−2.24 (17)	C3—O1—C6—C11	−119.50 (10)
F1—C1—C2—C3	178.34 (10)	C3—O1—C6—C7	64.48 (12)
C6—O1—C3—C2	30.14 (14)	C11—C6—C7—C8	2.07 (15)
C6—O1—C3—C4	−153.56 (9)	O1—C6—C7—C8	177.84 (9)
F2—C2—C3—O1	−1.91 (16)	C11—C6—C7—C7^i^	−176.48 (10)
C1—C2—C3—O1	177.56 (10)	O1—C6—C7—C7^i^	−0.71 (15)
F2—C2—C3—C4	−178.24 (9)	C6—C7—C8—C9	−1.77 (15)
C1—C2—C3—C4	1.24 (15)	C7^i^—C7—C8—C9	176.77 (10)
O1—C3—C4—F3	3.69 (14)	C7—C8—C9—C10	0.33 (16)
C2—C3—C4—F3	−179.66 (9)	C8—C9—C10—C11	0.92 (17)
O1—C3—C4—C5	−176.05 (9)	C7—C6—C11—C10	−0.88 (16)
C2—C3—C4—C5	0.61 (15)	O1—C6—C11—C10	−176.78 (9)
C1—N1—C5—F4	−179.01 (10)	C9—C10—C11—C6	−0.66 (16)
C1—N1—C5—C4	0.98 (17)		

Symmetry code: (i) −*x*+1, *y*, −*z*+3/2.
